# Nanocall: an open source basecaller for Oxford Nanopore sequencing data

**DOI:** 10.1093/bioinformatics/btw569

**Published:** 2016-09-10

**Authors:** Matei David, L J Dursi, Delia Yao, Paul C Boutros, Jared T Simpson

**Affiliations:** 1 Ontario Institute for Cancer Research, Toronto, Canada; 2 Department of Pharmacology and Toxicology, University of Toronto, Toronto, Canada; 3 Department of Medical Biophysics, University of Toronto, Toronto, Canada; 4 Department of Computer Science, University of Toronto, Toronto, Canada

## Abstract

**Motivation:**

The highly portable Oxford Nanopore MinION sequencer has enabled new applications of genome sequencing directly in the field. However, the MinION currently relies on a cloud computing platform, Metrichor (metrichor.com), for translating locally generated sequencing data into basecalls.

**Results:**

To allow offline and private analysis of MinION data, we created Nanocall. Nanocall is the first freely available, open-source basecaller for Oxford Nanopore sequencing data and does not require an internet connection. Using R7.3 chemistry, on two *E.coli* and two human samples, with natural as well as PCR-amplified DNA, Nanocall reads have ∼68% identity, directly comparable to Metrichor ‘1D’ data. Further, Nanocall is efficient, processing ∼2500 Kbp of sequence per core hour using the fastest settings, and fully parallelized. Using a 4 core desktop computer, Nanocall could basecall a MinION sequencing run in real time. Metrichor provides the ability to integrate the ‘1D’ sequencing of template and complement strands of a single DNA molecule, and create a ‘2D’ read. Nanocall does not currently integrate this technology, and addition of this capability will be an important future development. In summary, Nanocall is the first open-source, freely available, off-line basecaller for Oxford Nanopore sequencing data.

**Availability and Implementation:**

Nanocall is available at github.com/mateidavid/nanocall, released under the MIT license.

**Supplementary information:**

Supplementary data are available at *Bioinformatics* online.

## 1 Introduction

The MinION produced by Oxford Nanopore Technologies (ONT) is a highly portable, third-generation sequencing instrument, comparable in size to a cell phone. The small form factor makes the MinION particularly suitable for sequencing experiments performed in remote locations (Quick *et al.*, 2016). However, the MinION relies on the cloud computing platform Metrichor (metrichor.com) for *basecalling*, i.e. translating the low-level, locally generated sequencing data into DNA sequence reads. In a multi-site evaluation of the MinION using the SQK-MAP005 sequencing kit, Ip *et al.* (2015) obtained an average yield of ∼115 Mbp of ‘2D’ sequence data. This is encoded by Metrichor in more than 50 Gb (basecalls and original events). One can easily envisage a setting in which the scarcity of internet access can limit the effectiveness of using a MinION for sequencing. Furthermore, the Metrichor source code is only available under a restrictive proprietary license, and we believe an open source basecaller would be valuable to the development community.

To address these limitations, we created and introduce here Nanocall, a basecaller for MinION sequencing data. Nanocall provides an offline alternative to Metrichor. In this sense, Nanocall has one important shortcoming compared to Metrichor, in that it performs strand-specific basecalls (‘1D’, in Metrichor terminology), but it does not attempt to integrate the information from complementary strands (‘2D’). Still, we envisage multiple use cases for Nanocall: (i) as part of event-level pile-up analysis pipelines that only require approximate mapping locations; (ii) in situations where internet access is limited, such as remote sequencing; (iii) as a rapid quality assessment check (e.g. that the correct sample was sequenced) prior to basecalling with Metrichor, allowing for real-time quality control; and (iv) as an open source platform for testing new basecalling ideas and models.

ONT has recently introduced a new sequencing pore, R9. All data used in this paper is based on the previously available R7.3 sequencing pore using SQK-MAP006 sequencing kits.

## 2 Background

### 2.1 Sequencing overview

Informally, the MinION sequencer works as follows. First, DNA is sheared into fragments of 8–20 kbp and adapters are ligated to either end of the fragments. The resulting DNA fragments pass through a protein embedded in a membrane via a nanometre-sized channel (this protein is the ‘nanopore’). A single strand of DNA passes through the pore; the optional use of a hairpin adapter at one end of the fragment allows the two strands of DNA to serially pass through nanopore, allowing two measurements of the fragment. In ONT terminology, the first strand going through the nanopore is the *template*, and the second is the *complement*. As a DNA strand passes through the pore it partially blocks the flow of electric current through the pore. The flow of current is sampled over time which is the observable output of the system. The central idea is that the single-stranded DNA product present in the nanopore affects the current in a way that is strong enough to enable decoding the electric signal data into a DNA sequence. This process, called *basecalling*, takes as input a list of current measurements, and produces as output a list of DNA bases most likely to have generated those currents.

### 2.2 Segmentation

The first part of the decoding process is to segment the sampled current measurements into blocks. The nanopore current measurements are taken at regular time intervals, but the threading of the single-stranded DNA product through the nanopore is a stochastic process controlled by biological enzymes. The segmentation process takes as input the list of current measurements and produces a list of *events*, each consisting of: *start*start_*i*_, *length*length_*i*_, *mean*mean_*i*_ and *standard deviation*stdv_*i*_. Ideally, each event corresponds to a different DNA context found inside the nanopore, and consecutive events correspond contexts differing by exactly one base. In practice, the segmentation process is noisy, so the event sequence will inevitably contain ‘stays’ (consecutive events corresponding to the same context) and ‘skips’ (consecutive events corresponding to contexts different by more than one base). The segmentation process is performed locally by the MinKNOW software running on the host computer.

### 2.3 Pore models

ONT provide *pore models* describing the events that are expected to be observed for various DNA contexts. For the SQK-MAP006 data used in this paper, the models use DNA sequences of length 6 (‘6-mers’) as context, and for each 6-mer, they contain the *mean μ_k_* and *standard deviation σ_k_* of a Gaussian distribution modelling the *event mean*, and the *mean η_k_* and *standard deviation γ_k_* of an Inverse Gaussian modelling the *event standard deviation*. These pore models are included in the basecalled FAST5 files produced by Metrichor, and they depend only on the chemistry being used, not on the specific sample. There are currently three models in use, one for the template strand and two for the complement strand.

### 2.4 Scaling parameters

As a further complication, the current measurements have slightly different characteristics between nanopores, and between the times when they are taken by the same nanopore. To account for these variations, Metrichor uses a set of read- and strand-specific scaling parameters: shift, scale, drift, var, scale’ and var’. Using these parameters, if event *i* corresponds to context *k*, then:

(1) $$\begin{array}{cc} \mathrm{mea}n_{i} & ∼N(\mathrm{scale}\cdot \mu_{k}+\mathrm{shift}+\mathrm{drift}\cdot \mathrm{star}t_{i},\\ & \;\;{\left(\mathrm{var}\cdot \sigma_{k}\right)}^{2}), \end{array}$$

(2) $$\begin{array}{cc} \mathrm{std}v_{i} & ∼\text{IG}(\mathrm{scale}\prime \cdot \eta_{k},\mathrm{var}\prime \cdot \gamma_{k}). \end{array}$$

### 2.5 Basecalling

The core of the decoding process is the basecalling step, performed in the cloud by Metrichor, which infers the DNA sequence most likely to have produced the observed event sequence. Metrichor uses a Hidden Markov Model (HMM) for basecalling data, where the hidden state corresponds to the DNA context present in the nanopore, and where the pore models are used to compute emission probabilities. HMMs have been used before to model Oxford Nanopore data (Loman *et al.*, 2015; Schreiber and Karplus, 2015; Szalay and Golovchenko, 2015; Timp *et al.*, 2012). We describe our approach to basecalling in the following section.

## 3 Methods

Our motivation behind Nanocall is to offer an offline alternative to Metrichor, and since the initial signal segmentation step is performed locally, we do not seek to replace it. As such, the input to Nanocall consists of a set of segmented event sequences, stored in ONT-specific FAST5 files. Nanocall processes each input file separately as follows. It begins by splitting the template and complementary strands into separate event sequences when a hairpin is found. Next, it estimates the pore model scaling parameters. Optionally, Nanocall can perform several rounds of training to update the scaling parameters using the Expectation Maximization algorithm, and also to update the state transition parameters using the standard Baum–Welch algorithm (Baum, 1972). Finally, Nanocall performs standard Viterbi decoding of the path through the hidden states, where the state is the 6-mer in the nanopore. Nanocall can also be given a set of state transition probabilities and a set of pore models, but these are optional, and if they are not specified, sensible defaults are used. The details of the individual steps are given below. For a reference on standard HMM algorithms, see Durbin *et al.* (1998).

### 3.1 State transitions

The state transitions are prior probabilities of moving from one state to another state. The default state transitions are computed based on two parameters: the ‘stay’ probability *p*_stay_ and the ‘skip’ probability *p*_skip_. The former, *p*_stay_, is the probability that two consecutive events are emitted from the same context/state. This corresponds to a segmentation error where an erroneous event break was introduced. The latter, *p*_skip_, is the probability that two consecutive events are emitted from states that differ by more than one kmer shift. This corresponds to either a segmentation error or a sequencing error (i.e. the DNA moved too quickly through the pore to register a detectable event) where one or more events were lost. A slight complication is that, by increasing the number of skips, there is always more than one way of going from any one state to any other. For example, ACGTGT can be followed by GTGTAC using either one or three skips. Our computation of the state transitions takes this into account:

(3) $$\begin{array}{ll} \tau (k_{1},k_{2})=\; & \delta_{k_{1}=k_{2}}\cdot p_{\text{stay}}\\ & +\delta_{\text{suffix}(k_{1},5)=\text{prefix}(k_{2},5)}\cdot p_{\text{step}}\cdot \frac{1}{4}\\ & +\sum_{i=2}^{5}\delta_{\text{suffix}(k_{1},6-i)=\text{prefix}(k_{2},6-i)}\cdot p_{\text{skip1}}{}^{i-1}\cdot \frac{1}{4^{i}}\\ & +{\sum_{i>5}p_{\text{skip1}}}^{i-1}\cdot \frac{1}{4^{6}}. \end{array}$$

Here, δ is the standard indicator function; prefix(k,i)/suffix(k,i) are the prefix/suffix of *k* of length *i*; $p_{\text{step}}:=(1-p_{\text{stay}}-p_{\text{skip}})$; and $p_{\text{skip}1}=p_{\text{skip}}/(1+p_{\text{skip}})$ corresponds to the probability of exactly one skip.

To speed up computation, for both Forward-Backward (used during training) and Viterbi (used during basecalling) algorithms, we disregard transitions between states corresponding to more than one skip. Thus, each state has at most 21 neighbours: itself, 4 at distance 1, and 16 at distance 2. The downside of this heuristic is that a real 2-step skip will probably be mislabelled, leading to basecalling errors.

By default, Nanocall uses $p_{\text{stay}}=.1$ and $p_{\text{skip}}=.3$. When transition parameter training is enabled (see Supplement), the values of *p*_stay_ and *p*_skip_ are updated individually for each strand of each read, using the Baum-Welch algorithm.

### 3.2 Pore models

Nanocall is designed to work by default with the (6-mer based) pore models provided by Metrichor. These are three pore models, one for the template strand and two for the complement strand. Emission probabilities are calculated by multiplying the probability density from the Gaussian and Inverse Gaussian distributions that model the event mean and event standard deviation, respectively. This is done after the models are scaled (see Scaling below.) Nanocall can also run with user-provided pore models, though changing the kmer size requires recompilation.

### 3.3 Strand separation

The segmented list of events available in pre-Metrichor FAST5 files does not always contain markers delimiting the template and complement strands. To deal with this, Nanocall implements a simple heuristic for separating the strands. The core idea here is that the ‘hairpin’ adapter connecting the strands (in the single-stranded DNA product being threaded through the nanopore) contains abasic DNA, that is, DNA backbone lacking DNA bases. This abasic DNA generates a specific signal in the event sequence.

In the first step, we use a heuristic to detect the *abasic current level*. In general, the currents corresponding to regular DNA are in the range 50–90 pA, and those corresponding to abasic DNA are higher than 100 pA. However, the exact levels are affected by the shift and scale parameters, which are not known *a priori*. For that reason, Nanocall uses a heuristic to estimate abasic current level. Specifically we take the current level larger than 99% of all event levels in the read, and add 5 pA.

In the second step, Nanocall looks for islands of 5 or more consecutive abasic current measurements. Then, islands within 50 bp of each other are merged. Next, Nanocall selects the island closest to the middle of the event sequence. If this island is within the middle third of the entire event sequence it is used to separate the events corresponding to the two strands. If on the other hand this island is outside of the middle third of the event sequence, Nanocall gives up trying to separate the strands and attempts to basecall the entire event sequence as a template strand.

### 3.4 Scaling

Pore model scaling parameter estimation is a crucial part of the basecalling process. Since accurate scaling dominates the running time, Nanocall presents the user with several scaling options.

In all cases, Nanocall initializes the scaling parameters using a crude ‘Method of Moments’ approach, that matches the first two moments of the distribution of pore model kmer means to the first two moments of the distribution of (a subset of) sequenced event means. This approach relies on the assumption that the states producing the observed events are sampled uniformly at random, which is clearly not the case. However, for event sequences that are long enough, this method provides a reasonable base setting. This method only estimates 2 of the 6 scaling parameters discussed in the Background section: shift and scale. The remaining 4 are left with default values (0 for additive terms, 1 for multiplicative factors). The MoM scaling is the fastest, but also the least accurate. Since the other available scaling options dominate the total runtime, Nanocall can be instructed to use only MoM scaling, which will result in the fastest operation (command line option --no-train).

Beyond MoM, Nanocall offers the option to train the pore model scaling parameters by performing several rounds of an Expectation Maximization (EM) algorithm. The training stops either when the maximum allowed number of rounds is reached (by default 10 for single-strand scaling/20 for double-strand scaling, see below), or when the improvement in likelihood drops below a certain threshold (by default, a multiplicative factor, *e*). Each training round proceeds as follows. First, the ‘E’ step consists of running the Forward-Backward algorithm to compute state posterior probabilities on 2 subsets of the event sequence, extracted from the start and end of each strand. Next, the ‘M’ step updates all 6 scaling parameters with values that maximize the likelihood of the observed emissions given the probabilistic state assignments. More details about this update process are given in the Supplement.

Nanocall provides the user with the option of performing either single- or double-strand scaling. With single-strand scaling (option --single-strand-scaling), the 2 strands of each read are processed independently of each other. The motivation for double-strand scaling (option --double-strand-scaling, on by default) is to avoid potential overfitting of the two strands separately by constraining the scaling parameters to be the same across the read.

### 3.5 Viterbi decoding

After any optional training, Nanocall runs the Viterbi decoding algorithm to compute the most likely state sequence to have generated the observed event sequence. Then, the final base sequence is constructed by iteratively adding the minimum number of bases required to transition between consecutive states. Thus, for instance, if 2 consecutive states are ACTCTC and CTCTCA, the base sequence produced will be ACTCTCA, not ACTCTCTCA. In particular, the basecalled sequence will never contain a homopolymer repeat longer than the kmer size (6 bp) because of this heuristic and the fact that the state does not vary while such a sequence is being thread through the pore. However, repeats of sequences of length greater than 1 are still detectable. As a result of this heuristic, Nanocall reads will have systematic (non-random) errors around size-1 repeats.

When several pore models are applicable to the same strand, as is the case with the default models and the complement strand, Nanocall will select which model to use during training if (training is performed, and) the Forward-Backward computed likelihood using one model is better than all other applicable models by a multiplicative factor of *e*^20^. If a model is not selected during training, the read strand is basecalled using all applicable models. Then, the model used for output will be the one with a higher joint probability of the most probable path through the HMM and the observations, found in the last cell of the Viterbi matrix Durbin *et al.* (1998).

### 3.6 Quality values

Nanocall does not produce base quality value estimates primarily because the individual base error rate is too high for base qualities to be informative. For this reason, the output is in fasta format, not fastq.

### 3.7 Evaluations

In our evaluations, we use BWA MEM with options ‘-x ont2d’ for mapping ONT reads to the reference (Li, 2013). A 1D read (Metrichor or Nanocall) is considered *mapped* if its BWA MEM mapping overlaps the mapping of the corresponding Metrichor 2D read. Otherwise, the 1D read is *mismapped* (i.e. it is not mapped, or mapped elsewhere). The *identity* of a mapped read is defined as the percentage of match columns in the alignment (i.e. matches divided by read bases aligned plus reference bases deleted). In our results, we only measure identity for reads where all 4 1D reads (2× Nanocall, 2× Metrichor 1D) are mapped.

### 3.8 Source code

Nanocall is written in C ++11 and its source code is available at github.com/mateidavid/nanocall, along with instructions for how to build it either in a standard UNIX environment, or as a Docker container. The analysis scripts used to generate the data presented in this paper are available at github.com/mateidavid/nanocall-analysis.

### 3.9 Data availability

The datasets used in this paper are available in the European Nucleotide Archive under accession numbers ERR1147227 (*E.coli* native), ERR1147229 (*E.coli* PCR), ERR1309550 (human native) and ERR1309553 (human PCR). The *E.coli* datasets were described at lab.loman.net/2015/09/24/first-sqk-map-006-experiment, as a follow-up to earlier work (Quick *et al.*, 2014).

## 4 Results

We ran Nanocall on four ONT datasets consisting of *E.coli*, PCR-amplified *E.coli*, human, and PCR-amplified human DNA. For each dataset, we used the first 10 000 reads labelled as ‘passing’ by Metrichor. A summary of the key dataset statistics is given in Table 1. This includes the efficiency of the Nanocall hairpin detection heuristic, as well as alignment characteristics for the various read types considered. The Nanocall runs in this table use the default parameters (double strand scaling, 2ss). Interestingly, both Metrichor and Nanocall show a small bias for insertion errors in template reads and for deletion errors in complement reads.

**Table 1:** Dataset summary

Dataset	Reads	Avg Events	Hairpin		Avg Length					Identity			Insertions				Deletions			
			N	MN	M2	M0	M1	N0	N1	M2	M	N	M0	M1	N0	N1	M0	M1	N0	N1
*E.coli*	10000	19160	.994	.904	9803	9701	9135	9173	8741	.861	.699	.681	.071	.055	.058	.044	.07	.097	.09	.121
*E.coli* PCR	10000	13448	.991	.929	6861	6837	6419	6458	6152	.874	.706	.688	.071	.053	.058	.042	.069	.095	.089	.118
Human	10000	11145	.892	.863	5977	5816	5538	5594	5780	.857	.688	.673	.051	.041	.042	.031	.09	.11	.107	.133
Human PCR	10000	8604	.961	.937	4377	4265	4083	4074	3993	.858	.69	.676	.057	.045	.047	.038	.085	.104	.103	.122

Reads: number of ONT reads for which the Metrichor 2D read was mapped to the reference. Avg Events: average number of events. Hairpin N: fraction of reads for which Nanocall detects a hairpin. Hairpin MN: fraction of reads for which the Metrichor and Nanocall hairpins are within 100 events of each other. Avg Length: average basecalled read length for each read type: Metrichor 2D (M2), Metrichor template/complement (M0/M1), and Nanocall template/complement (N0/N1). Identity: alignment identity for Metrichor 2D, Metrichor 1D and Nanocall 1D reads. Insertions/Deletions: fraction of insertion/deletions for each 1D read type. The Nanocall runs in this table use the default options (double-strand scaling, 2ss).

For each dataset, we ran Nanocall using 5 different option sets: no training (option --no-train, labelled fast); single/double strand scaling (options --single-strand-scaling and --double-strand-scaling, labelled 1ss/2ss); with (default) and without transition parameter training (option --no-train-transitions, labelled nott). The results are given in Table 2. One conclusion supported by these runs is that the fast mode, with no training at all, seems very adequate for *E.coli* data, but much less so for human data. The difference between the two is the size and complexity of the genome, with *E.coli* eads being much easier to map.

**Table 2:** Performance of Nanocall

Dataset	Opts	Hpin	MCorr	NCorr	MIdn	NIdn	Speed*
*E.coli*	2ss-nott	.904	.962	**.942**	.699	.682	1106
	2ss			.941		.681	763
	1ss-nott			.941		.682	1655
	1ss			.938		**.683**	1217
	fast			.929		.673	**2834**
*E.coli* PCR	2ss-nott	.929	.978	**.959**	.706	**.69**	909
	2ss			.957		.688	614
	1ss-nott			.957		.689	1378
	1ss			.955		.69	974
	fast			.936		.68	**2858**
Human	2ss-nott	.863	.763	**.72**	.688	**.677**	747
	2ss			.703		.673	515
	1ss			.647		.672	839
	1ss-nott			.642		.675	1209
	fast			.441		.652	**3004**
Human PCR	2ss-nott	.937	.868	**.827**	.69	**.679**	569
	2ss			.823		.676	397
	1ss-nott			.785		.675	928
	1ss			.785		.675	647
	fast			.543		.655	**2888**

Options: fast: no training; 1ss/2ss: single/double strand scaling; nott: no transition parameter training. Hpin: fraction of reads where Metrichor and Nanocall hairpins are within 100 events of each other. M/N Corr: fraction of Metrichor 1D/Nanocall reads where the mapping of both strands overlaps the mapping of the Metrichor 2D read. M/N Idn: Metrichor 1D/Nanocall identity, for reads where all 5 mappings (1× Metrichor 2D, 2× Metrichor 1D, 2× Nanocall) are overlapping. Speed: Kbp per core-hour (*: measured separately for 1000 reads only, on a desktop computer with a 4-core Intel(R) Core(TM) i5-3570 CPU and 12GB of RAM). In bold face: best value in column, among options for that dataset

Another conclusion we were able to draw is that double strand scaling (2ss) performs better than single strand scaling (1ss). While the effect is minimal on *E.coli* data, it becomes quite pronounced on human data, where single strand scaling leads to an additional 4–6% reads being mismapped. When it comes to transition parameters, surprisingly, no training performs slightly better than training on human data: 2% on human native data, <1% on human PCR data. However, not training transition parameters makes the performance of a run much more dependent on the default transition parameters. For this reason, and in spite of the slight increase in mismapping rate, we decided on using double strand scaling with transition parameter training (2ss) as the Nanocall default.

Overall, Table 2 shows that the reads produced by Nanocall (2ss) are directly comparable to Metrichor 1D reads: Nanocall increases the mismapping rate by an additional ∼3% for *E.coli* data, and ∼6% on human data. Furthermore, Nanocall reads have similar, yet slightly smaller (∼1%), percent identity compared to Metrichor 1D reads.

**Fig. 1:**
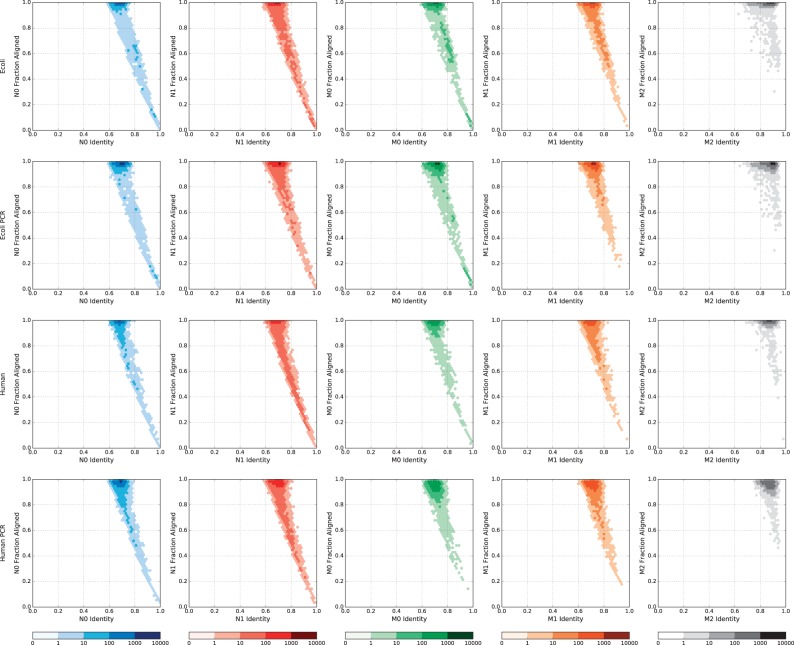
Fraction aligned versus identity using a base-10 logarithmic density plot, for all datasets (rows) and all read types (columns). Only showing reads where all 1D basecalls are mapped (Color version of this figure is available at *Bioinformatics* online.)

**Fig. 2:**
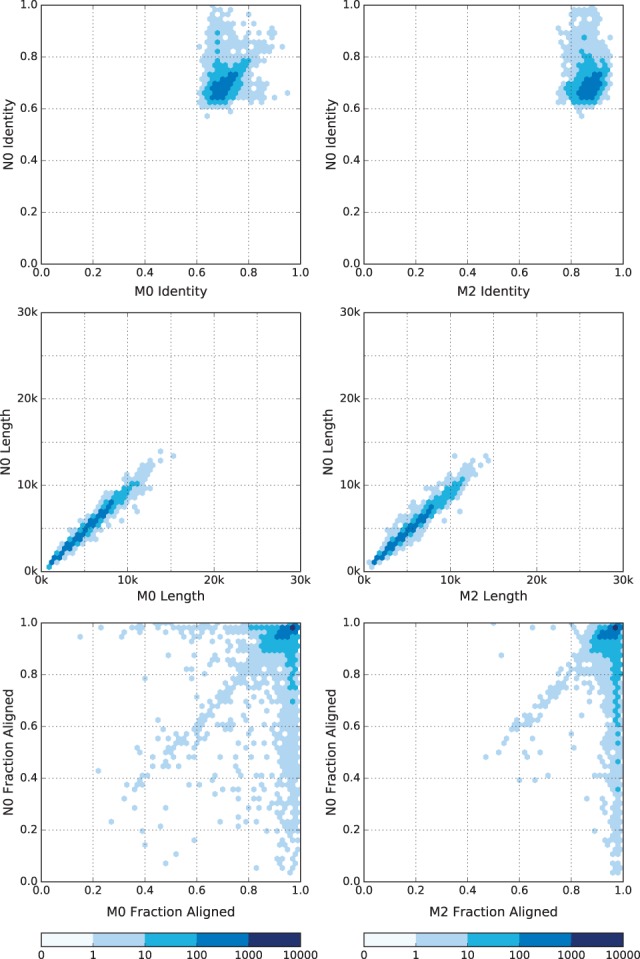
Comparison of Metrichor and Nanocall (2ss) mapped reads using a base-10 logarithmic density plot, for template reads from the human PCR dataset. We show Metrichor versus Nanocall: identity (row 1), read length (row 2) and fraction aligned (row 3). We compare Nanocall template reads with Metrichor template reads (column 1) and Metrichor 2D reads (column 2) (Color version of this figure is available at *Bioinformatics* online.)

The similarity between Nanocall and Metrichor 1D reads is further demonstrated in Figure 1, where we compare identity and fraction aligned, separately for all datasets and all read types, and Figure 2, where we compare identity, read length, and read fraction aligned between Nanocall template reads and Metrichor template and 2D reads from the human PCR dataset. The figures corresponding to complement reads, and to the other three datasets are very similar, and they are given in the Supplement.

**Fig. 3:**
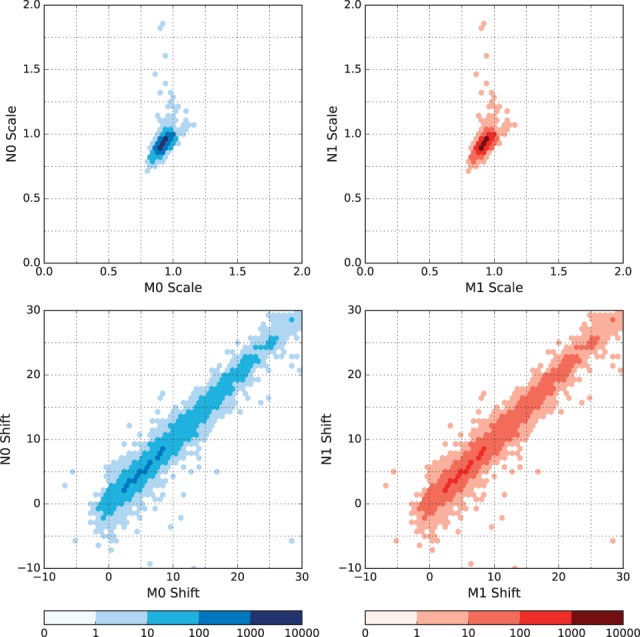
Nanocall (2ss) versus Metrichor scaling parameters for mapped reads using a base-10 logarithmic density plot, for the human PCR dataset. We show Metrichor vs Nanocall scale (row 1) and shift (row 2), for template reads (col 1) and complement reads (col 2). Only showing reads where both Metrichor and Nanocall detected a hairpin and picked the same complement model (Color version of this figure is available at *Bioinformatics* online.)

In Figure 3, we compare pore model scaling parameters scale and shift between Nanocall and Metrichor, separately for template and complement reads from the human PCR dataset, for reads where Nanocall detected a hairpin. In general, we see that Nanocall obtains very similar values to Metrichor. The figures corresponding to the remaining datasets are similar, and they are given in the Supplement.

**Fig. 4:**
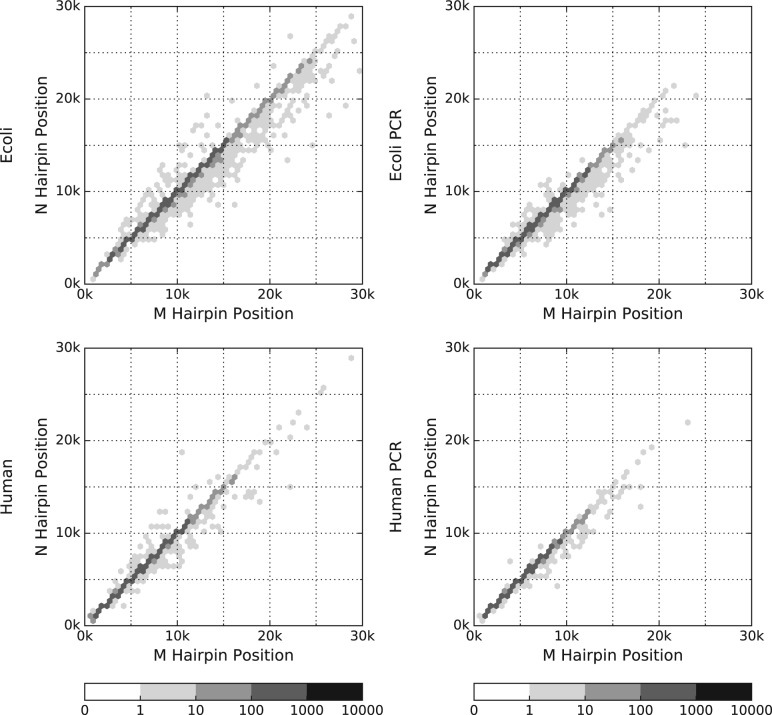
Nanocall versus Metrichor hairpin position using a base-10 logarithmic density plot, for reads where Nanocall detected a hairpin, for each dataset (Color version of this figure is available at *Bioinformatics* online.)

In Figure 4, we compare the hairpin positions detected by Metrichor and Nanocall in each dataset, for reads where Nanocall indeed detects a hairpin. We see that the simple heuristic employed by Nanocall works well enough in practice, generally producing results similar to Metrichor.

To quantify the effects of the default transition parameters, we used 1000 human PCR reads, and ran Nanocall in double strand scaling mode without transition parameter training (2ss-nott), using several parameter values: $p_{\text{stay}}\in \{.09,.1^{*},.11,.12\}$ and $p_{\text{skip}}\in \{.22,.24,.26,.28,.3^{*},.32\}$ (* denotes the default). The results of these runs are given in Table 3, showing that, while *p*_stay_ and *p*_skip_ do affect mappability, their influence is quite limited: a difference of <2% in mappability between all runs. Note: we expect *p*_stay_ and *p*_skip_ to have even smaller effect when transition parameter training is enabled (2ss).

**Table 3:** Influence of default transition parameters *p*_stay_ and *p*_skip_

*p*_stay_	*p*_skip_	MCorr	NCorr	MIdn	NIdn
.10	.24	.852	**.822**	.689	.679
.12	.28		.818		.679
.12	.24		.818		.679
.11	.22		.818		.679
.12	.22		.817		.679
.11	.24		.817		.679
.12	.30		.815		.679
.12	.26		.815		.679
.11	.26		.815		**.68**
.09	.26		.815		.679
.09	.24		.815		.679
.12	.32		.814		.679
.10	.30		.814		.679
.10	.22		.814		.679
.09	.22		.814		.679
.11	.28		.813		.679
.11	.32		.812		.679
.10	.32		.812		.679
.09	.28		.812		.679
.10	.28		.811		.679
.10	.26		.811		.68
.11	.30		.81		.679
.09	.32		.805		.679
.09	.30		.804		.679

All runs on 1000 human pcr reads, with double strand scaling with no transition parameter training (2ss-nott). Other columns: see Table 2.

We also studied the effects of perturbing the parameters that control training: the number of events used (default 100), the maximum number of rounds per strand (default: 10), and the minimum improvement in fit (default: additive term of 1.0 in log space, corresponding to multiplicative term *e*). The results are given in Table 4. Clearly, increasing the number of events used per strand (nume) directly improves mappability, but also decreases speed. Increasing the maximum number of training rounds (maxr) has the same effect, but improvements in mappability are lost beyond 10 rounds. The effect of the minimum fit improvement (minp) on mappability and even speed is harder to quantify.

**Table 4:** Influence of parameters that control training

Param	Val	MCorr	NCorr	MIdn	NIdn	Speed
nume	250	.852	**.821**	.689	**.678**	313
nume	200		.811		.676	397
minp	1.0		.811		.676	396
maxr	10		.811		.676	396
maxr	20		.81		.676	394
maxr	15		.81		.676	395
maxr	5		.81		.675	450
minp	1.5		.809		.675	445
minp	2.0		.805		.675	485
minp	.5		.801		.677	333
maxr	4		.8		.674	513
nume	150		.795		.675	530
maxr	3		.787		.673	639
nume	100		.767		.671	752
maxr	2		.764		.671	866
nume	50		.722		.668	**1220**

Runs on 1000 human PCR reads, using double strand scaling mode with transition parameter training (2ss). nume*x*: use *x* events used per strand; maxr*y*: maximum of *y* training rounds per strand; minp*z*: minimum fit improvement of *z* in log space. Other columns: see Table 2.

## 5 Conclusion

In this work we presented Nanocall, an open source, MIT licensed basecaller for data produced by Oxford Nanopore MinION instruments. Nanocall uses some simple heuristics for splitting the sequence of events (current levels) into strands, it models the events using a hidden Markov model where the states are the kmers being sequenced, it optionally scales the pore model emissions using several rounds of Expectation Maximization based on posteriors computed with Forward–Backward, and it produces basecalls by running Viterbi.

Overall, Nanocall produces reads comparable in mappability and quality to Metrichor 1D reads with ∼68% identity. As an important technical difference from Metrichor, with Nanocall we found that double-strand pore model scaling seems to work better than single-strand scaling, suggesting that the latter might lead to model overfitting.

## Supplementary Material

Supplementary Data
