# Supplementary material for: Nanocall: an open source basecaller for Oxford Nanopore sequencing data
Source: Bioinformatics. 2016 Sep 10;33(1):49–55. doi: 10.1093/bioinformatics/btw569 (PMC5408768; doi:10.1093/bioinformatics/btw569)
Supplement: Supplementary Data [file btw569_supp.zip › btw569_Supp1.pdf]

Nanocall Figures

Matei David, L.J. Dursi, Delia Yao, Paul C. Boutros, and Jared T. Simpson

July 11, 2016

Abstract

Full figures for the Nanocall analysis.

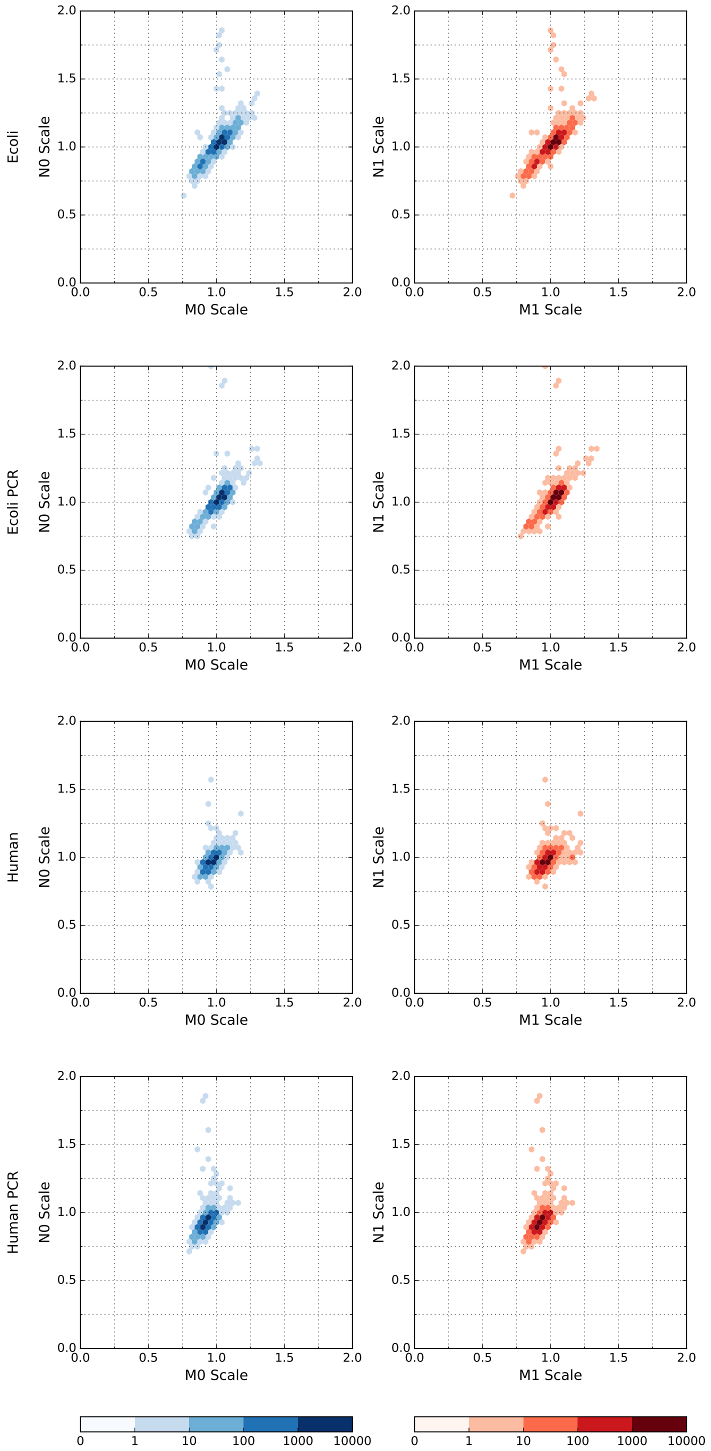

Figure 1: Nanocall vs Metrichor `scale`.

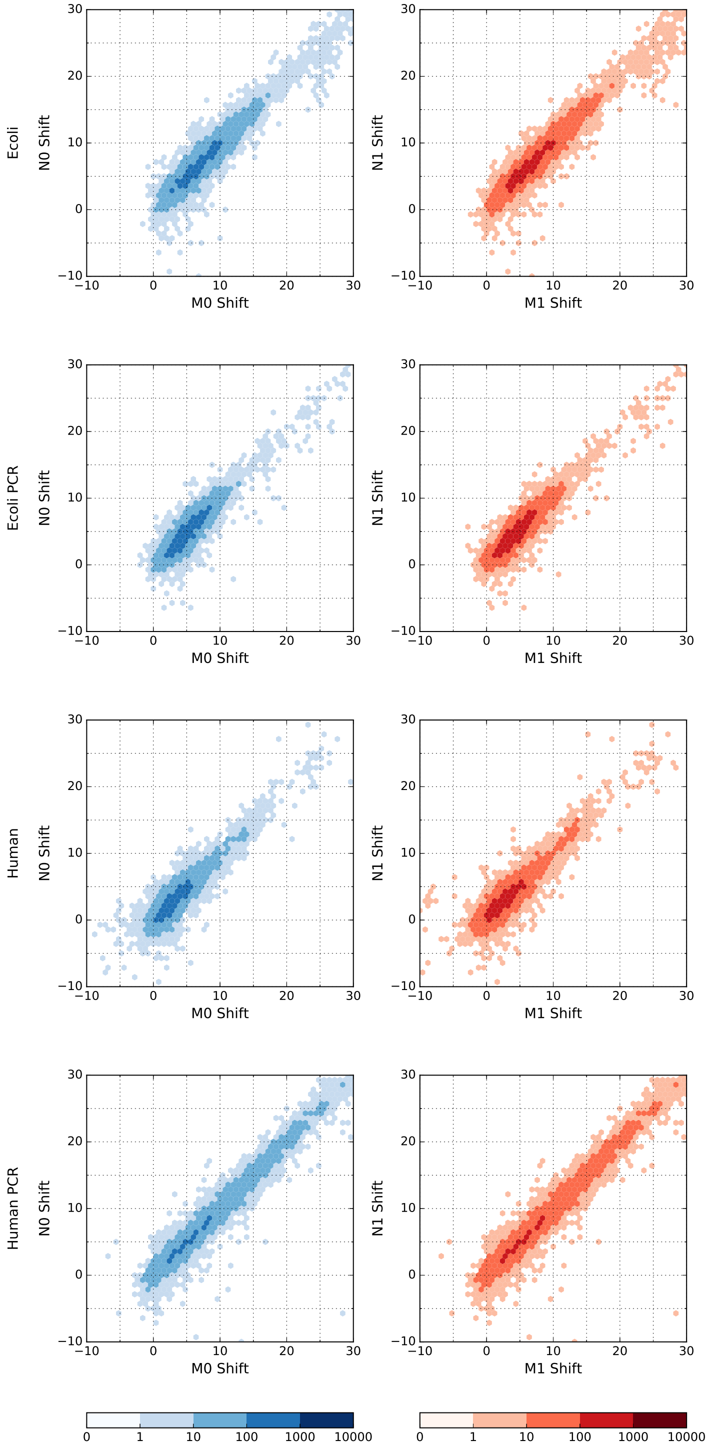

Figure 2: Nanocall vs Metrichor `shift`.

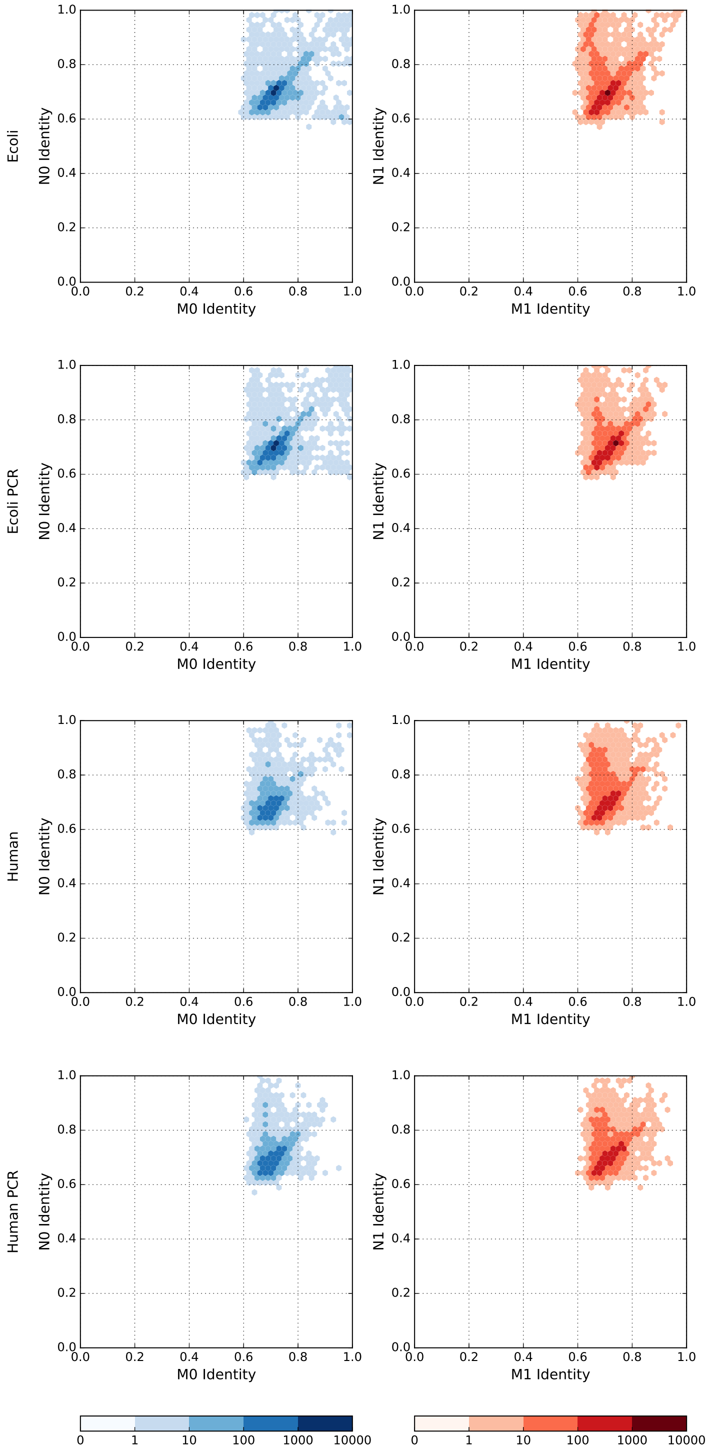

Figure 3: Nanocall vs Metrichor (same strand) read mapping identity.

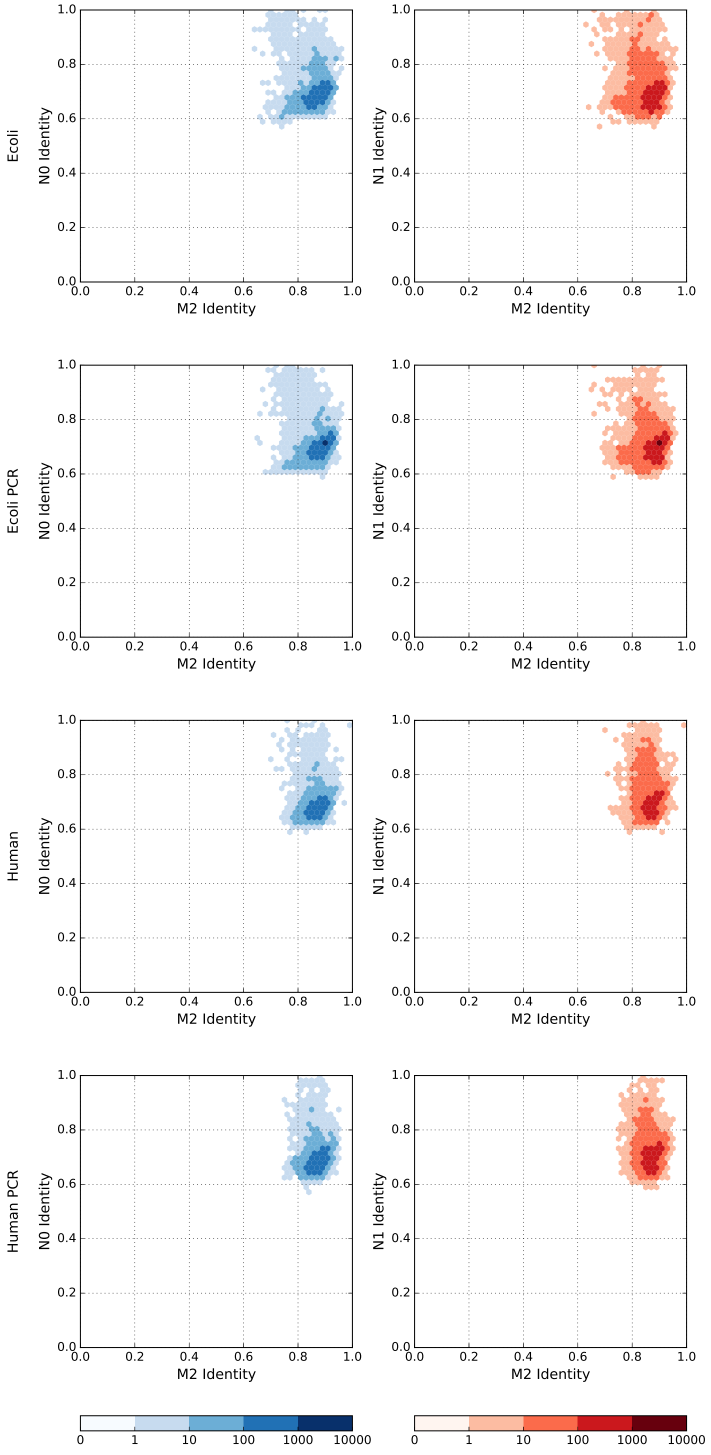

Figure 4: Nanocall vs Metrichor 2D read mapping identity.

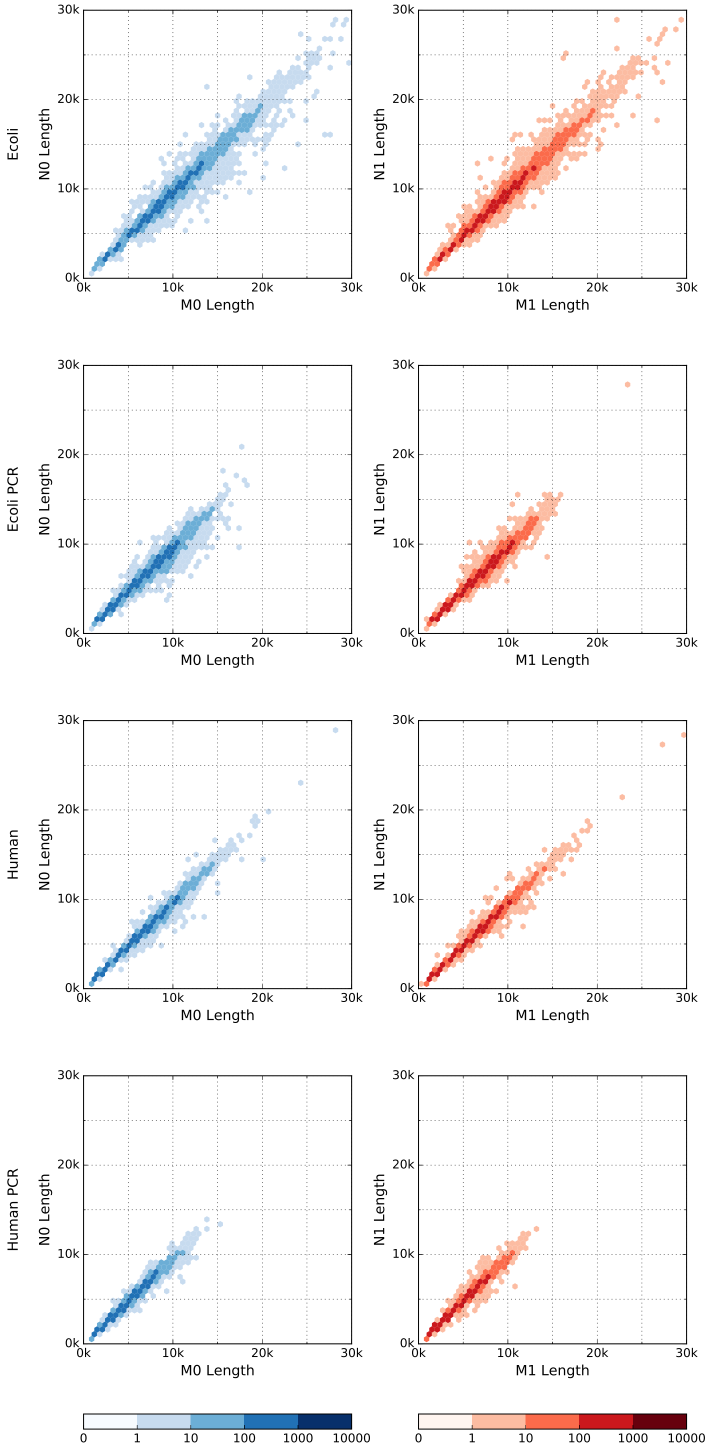

Figure 5: Nanocall vs Metrichor (same strand) read length.

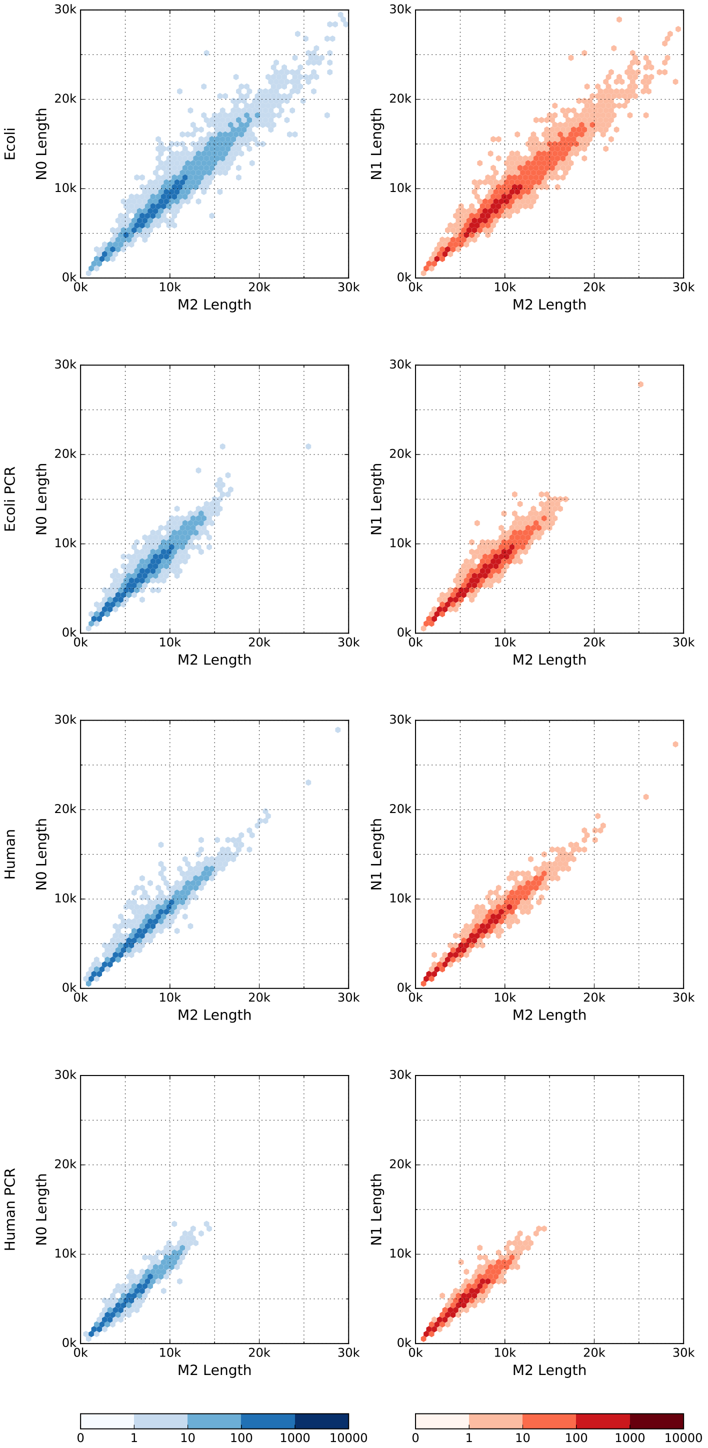

Figure 6: Nanocall vs Metrichor 2D read length.

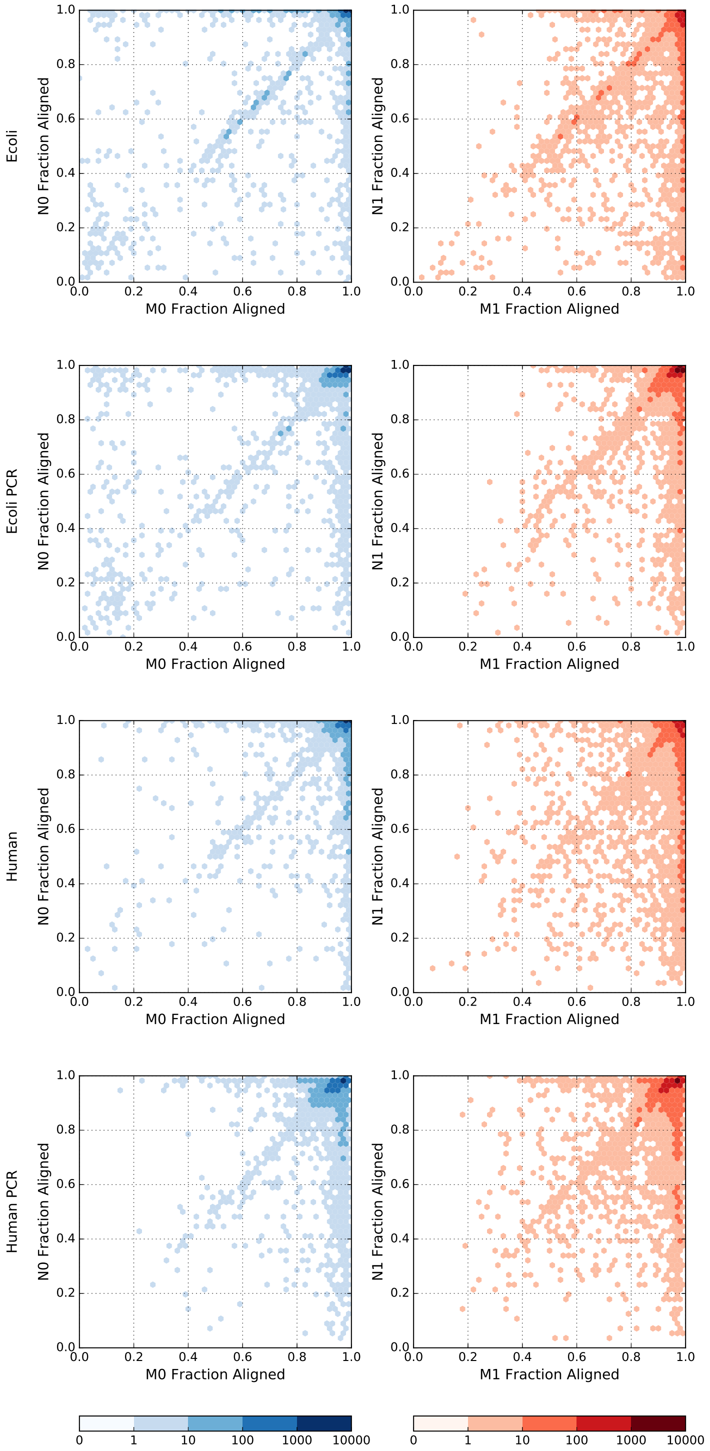

Figure 7: Nanocall vs Metrichor (same strand) read fraction aligned.

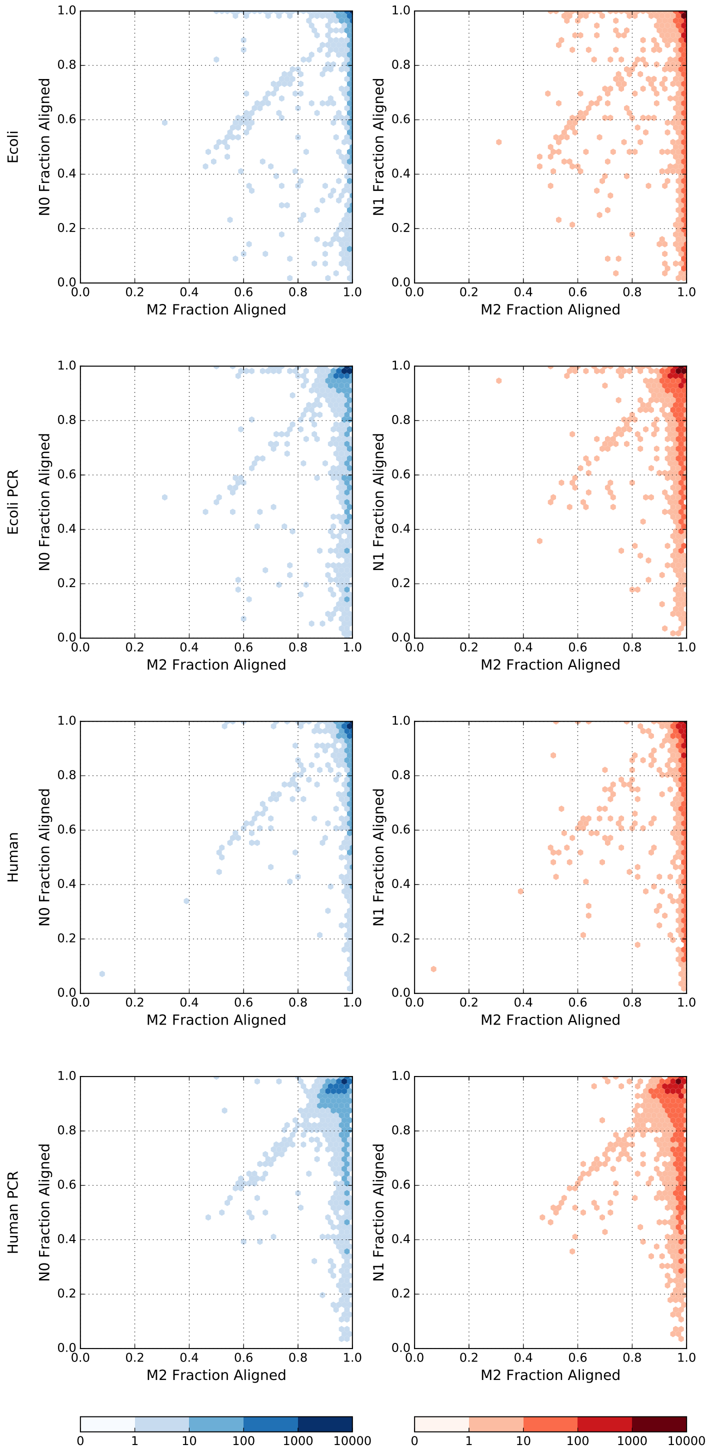

Figure 8: Nanocall vs Metrichor 2D read fraction aligned.

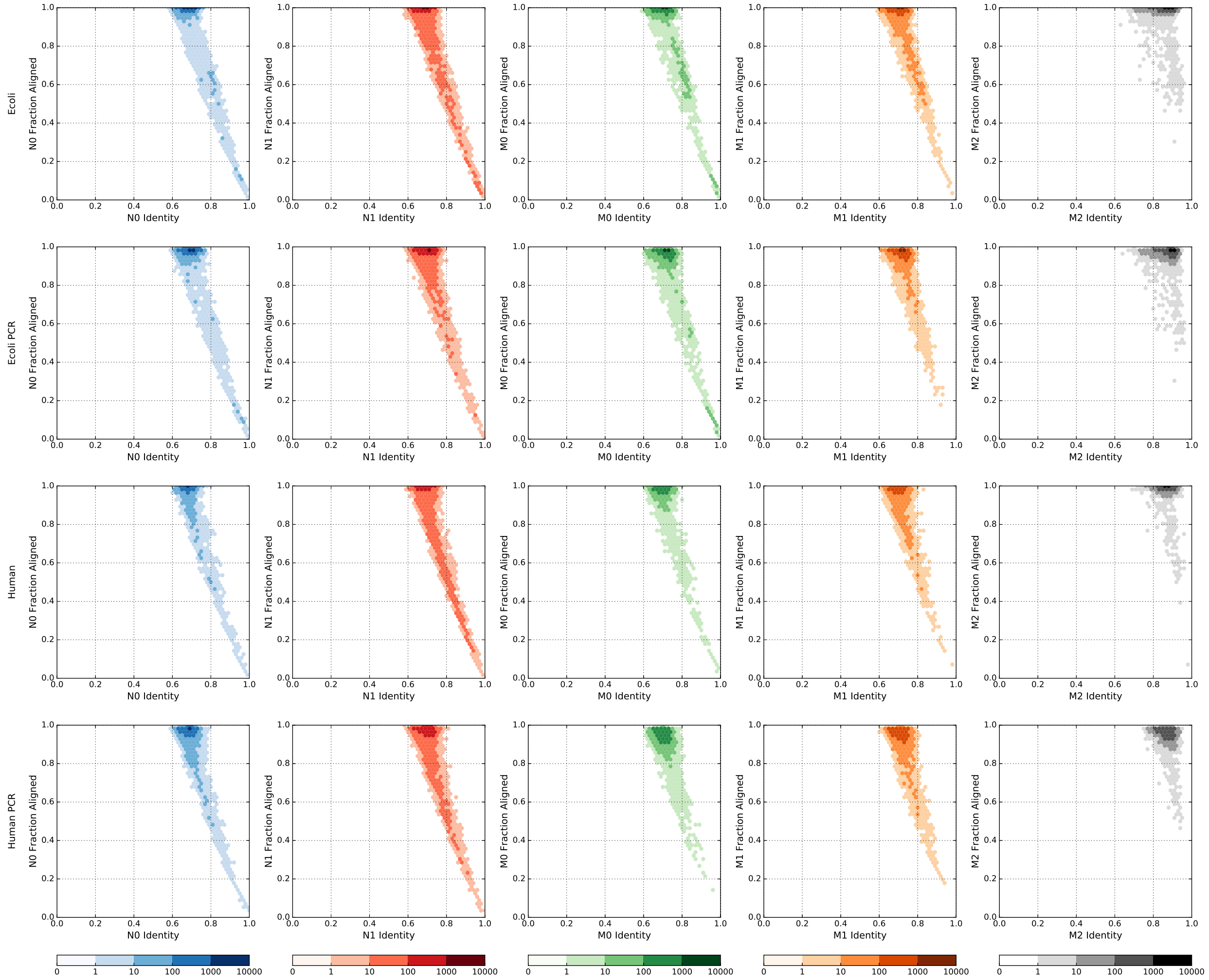

Figure 9: Nanocall vs Metrichor read mapping identity vs read read fraction aligned.

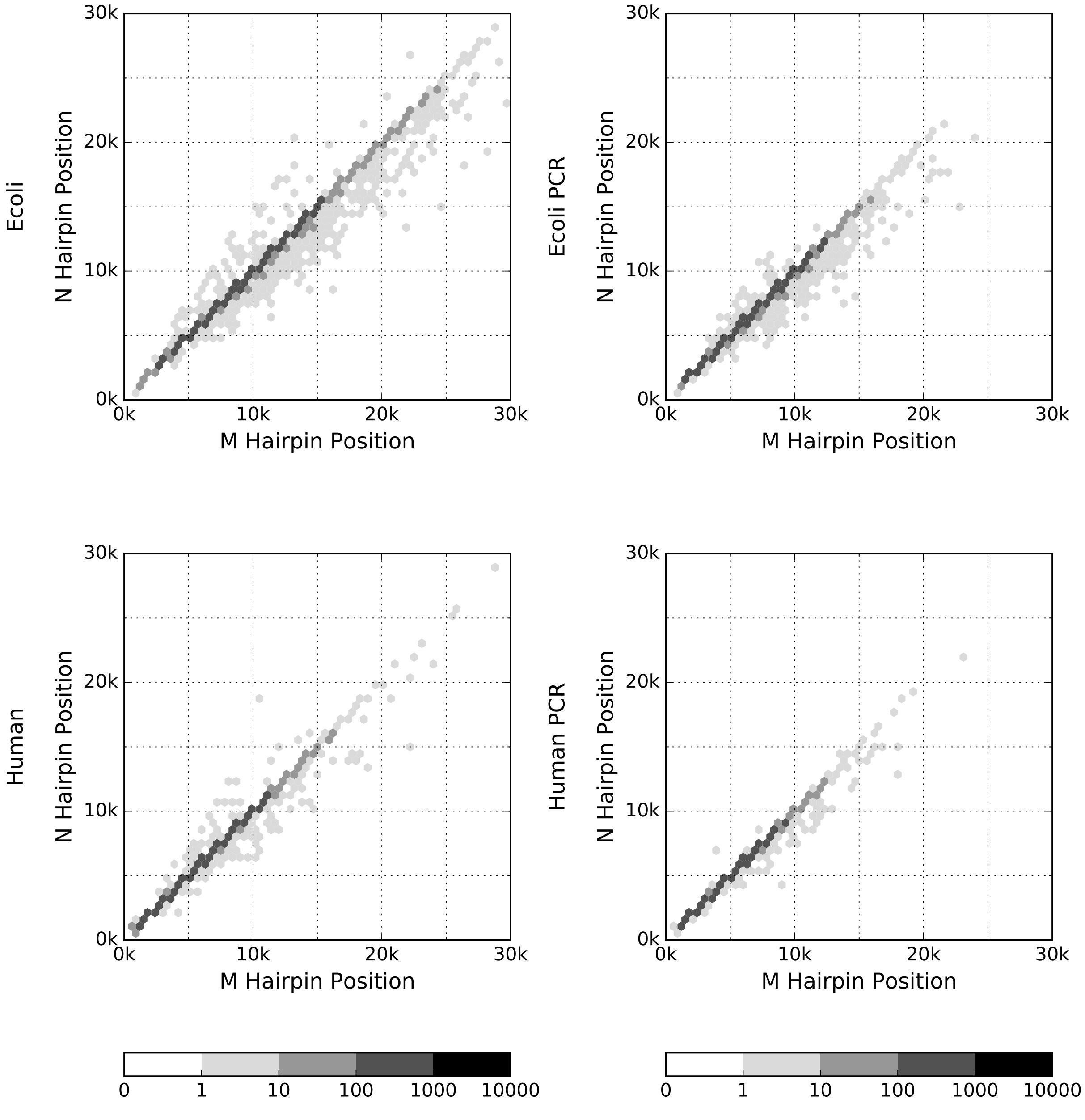

Figure 10: Nanocall vs Metrichor hairpin location.
