# Supplementary material for: Nanocall: an open source basecaller for Oxford Nanopore sequencing data
Source: Bioinformatics. 2016 Sep 10;33(1):49–55. doi: 10.1093/bioinformatics/btw569 (PMC5408768; doi:10.1093/bioinformatics/btw569)
Supplement: Supplementary Data [file btw569_supp.zip › btw569_Supp2.pdf]

Nanocall Training

Matei David, L.J. Dursi, Delia Yao, Paul C. Boutros, and Jared T. Simpson

April 4, 2016

Abstract

This document describes the updates to the pore model scaling parameters and the state transition parameters computed by Nanocall in one training round.

1 Introduction

When training is enabled, Nanocall performs several training rounds that update both the pore model scaling parameters (*shift*, *scale*, *drift*, *var*, *scale_sd*, *var_sd*) and the state transition parameters (p_{stay}, p_{skip}). Nanocall can use either two sets of pore model scaling parameters (with `--single-strand-scaling`) or a single common set (with `double-strand-scaling`). In either case, Nanocall will use separate per-strand state transition parameters. During each round, Nanocall processes 2 event subsequences (of total length controlled by `--scaling-num-events`, by default 200) from the start and end of either one or both strands. First, Nanocall runs the Forward-Backward algorithm separately on each subsequence using the current parameters, obtaining posteriors for each event and each state, and also the probability of the data, which we refer to as *fit*. Next, Nanocall updates the pore model scaling parameters as described in Section 3. Finally, Nanocall updates the transition parameters as described in Section 4.

Training stops either when a maximum number of rounds is reached (controlled by `--scaling-max-rounds`, by default 10 for single-strand scaling, and 20 for double-strand scaling), or when the fit improvement from the previous round drops below a certain threshold (controlled by `--scaling-min-progress`, default 1.0 in logspace). Note that the fit in fact represents the probability of the data using the previous round's parameters. (We believe it would have been wasteful to include an additional Forward-Backward run only for computing the fit with the last set of parameters.)

2 Notation

We use the following notation:

n Number of events.

i Index over events, so $i \in \{1, \ldots, n\}$.

m Number of states, so $m = 4^6$ if using 6-mers.

j Index over states, so $j \in \{1, \ldots, m\}$.

x_i Observed mean for event i.

y_i Observed stdv for event i.

t_i Measurement time for event i.

μ_j Model level mean for state j.

σ_j Model level stdv for state j.

η_j Model spread mean for state j.

γ_j Model spread stdv for state j.

λ_j Model spread (IG) shape for state j.

(a, b, c, d, v, u) Scaling parameters to compute, corresponding to, in order: *shift*, *scale*, *drift*, *var*^{2}, *scale_sd*, and *var_sd*.

$p_{i,j}$ Probability event i was emitted from state j.

$f_{i,j}$ Gaussian pdf for emitting event level i from state j.

$g_{i,j}$ Inverse Gaussian pdf for emitting event stdv i from state j.

(A, B) For matrices A and B with the same number of rows, (A, B) is the matrix containing A on the left and B on the right.

$(A; B)$ For matrices A and B with the same number of columns, $(A; B)$ is the matrix containing A on top and B on the bottom.

$A^{(k)}$ Is the column $(A; \ldots; A; \ldots; A)$ with A repeated k times.

x The column vector of x_i.

t The column vector of t_i.

suffix(k, i) The last i bases in kmer k.

prefix(k, i) The first i bases in kmer k.

3 Pore Model Scaling Parameters

The training procedure detailed in this section was originally described in the ONT internal document [1]. Our purpose here is first to clarify some of the original notation, and second to provide more details for the quantities computed in the Nanocall source code.

3.1 Gaussian Parameters

We want to compute the Gaussian scaling parameters (a, b, c, d) that maximize the log likelihood of the observed measurements:

$$\begin{aligned}
\log f_e &= \sum_{i,j} p_{i,j} \log f_{i,j} \\
&= \sum_{i,j} p_{i,j} \left(-\frac{(x_i - \mu'_{i,j})^2}{2\sigma'^2_j} - \frac{\log 2\pi\sigma'^2_j}{2} \right) \\
&= -\left(\sum_{i,j} \frac{p_{i,j} \left(x_i - a - b\mu_j - ct_i\right)^2}{2d\sigma_j^2} \right) - \left(\sum_{i,j} \frac{p_{i,j} \log 2\pi d\sigma_j^2}{2} \right) \\
&= -\frac{1}{2d} \left(\sum_{i,j} w_{i,j} \left(x_i - a - b\mu_j - ct_i\right)^2 \right) - \frac{\log d}{2} \left(\sum_{i,j} p_{i,j} \right) - \left(\sum_{i,j} \frac{p_{i,j} \log 2\pi\sigma_j^2}{2} \right)
\end{aligned}$$

Where

$\mu'_{i,j}$ $:= a + b\mu_j + ct_i$.

σ'^2_j $:= d\sigma^2_j$.

$w_{i,j}$ $:= \frac{p_{i,j}}{\sigma^2_j}$.

Further note that:

- The third term is independent of the scaling factors, so we can ignore it.
- Likewise, we can ignore the 1/2 factor.
- $\sum_{i,j} p_{i,j} = n$, as $\forall i, \sum_j p_{i,j} = 1$.

Thus, we want to solve:

$$(\hat{a}, \hat{b}, \hat{c}, \hat{d}) = \text{argmax}_{(a,b,c,d)} - \frac{1}{d} \left(\sum_{i,j} w_{i,j} \left(x_i - a - b\mu_j - ct_i \right)^2 \right) - n \log d$$

3.1.1 First step

We first solve for (a, b, c) while keeping d constant. Then, our goal is to solve the weighted linear system:

$$(\hat{a}, \hat{b}, \hat{c}) = \text{argmin}_{(a,b,c)} \sum_{(i,j)} w_{i,j} \left(x_i - a - b\mu_j - ct_i \right)^2$$

This is an overdetermined system with nm equations (iterated over using the tuple (i, j)) and 3 coefficients. We write it in standard (wikipedia) form as follows. Let:

(i, j) Index over the nm equations. When using the matrix notation below, we let (i, j) iterate over i first, then over j. Thus, the order of equations is:

$$(1, 1), \ldots, (n, 1), \ldots, (1, j), \ldots, (n, j), \ldots, (1, m), \ldots, (n, m)$$

$\boldsymbol{y}$ is the $nm \times 1$ column vector $x^{(m)}$.

$\boldsymbol{X}$ is the $nm \times 3$ matrix:

$$\boldsymbol{X} := \left((1^{(n)}, \mu_1^{(n)}, t); \ldots; (1^{(n)}, \mu_j^{(n)}, t); \ldots; (1^{(n)}, \mu_m^{(n)}, t) \right)$$

$\boldsymbol{\beta}$ is the 3×1 column vector $(a; b; c)$.

$\boldsymbol{W}$ is the $nm \times nm$ diagonal matrix of weights:

$$\boldsymbol{W} := \text{diag}\left((w_{1,1}, \ldots, w_{n,1}), \ldots, (w_{1,j}, \ldots, w_{n,j}), \ldots, (w_{1,m}, \ldots, w_{n,m}) \right)$$

With the notation above,

$$\hat{\boldsymbol{\beta}} = \text{argmin}_{\boldsymbol{\beta}} \left\| \boldsymbol{W}^{1/2} (\boldsymbol{y} - \boldsymbol{X\beta}) \right\|^2$$

This system has the known solution:

$$\hat{\boldsymbol{\beta}} = \left(\boldsymbol{X}^T \boldsymbol{WX} \right)^{-1} \left(\boldsymbol{X}^T \boldsymbol{Wy} \right)$$

To reconcile this solution with the ONT document, let:

$\boldsymbol{X}_j$ is the j-th block in $\boldsymbol{X}$, of size $n \times 3$:

$$\boldsymbol{X}_j := \left(1^{(n)}, \mu_j^{(n)}, t\right)$$

$\boldsymbol{W}_j$ is the j-th diagonal block in $\boldsymbol{W}$, of size $n \times n$:

$$\boldsymbol{W}_j := \operatorname{diag}\left(w_{1,j}, \ldots, w_{n,j}\right)$$

Repeatedly using the fact that $(A, B) \cdot (C; D) = A \cdot C + B \cdot D$, observe that:

$$\begin{aligned}
\boldsymbol{X}^T \boldsymbol{W} \boldsymbol{X} &= \left(\boldsymbol{X}_1^T, \ldots, \boldsymbol{X}_j^T, \ldots, \boldsymbol{X}_m^T\right) \operatorname{diag}\left(\boldsymbol{W}_1, \ldots, \boldsymbol{W}_j, \ldots, \boldsymbol{W}_m\right) \left(\boldsymbol{X}_1; \ldots; \boldsymbol{X}_j; \ldots; \boldsymbol{X}_m\right) \\
&= \sum_j \boldsymbol{X}_j^T \boldsymbol{W}_j \boldsymbol{X}_j, \\
&= \sum_j \left(1^{(n)}, \mu_j^{(n)}, t\right)^T \operatorname{diag}\left(w_{1,j}, \ldots, w_{n,j}\right) \left(1^{(n)}, \mu_j^{(n)}, t\right) \\
\boldsymbol{X}^T \boldsymbol{W} \boldsymbol{y} &= \left(\boldsymbol{X}_1^T, \ldots, \boldsymbol{X}_j^T, \ldots, \boldsymbol{X}_m^T\right) \operatorname{diag}\left(\boldsymbol{W}_1, \ldots, \boldsymbol{W}_j, \ldots, \boldsymbol{W}_m\right) \left(x; \ldots; x; \ldots; x\right) \\
&= \sum_j \boldsymbol{X}_j^T \boldsymbol{W}_j x \\
&= \sum_j \left(1^{(n)}, \mu_j^{(n)}, t\right)^T \operatorname{diag}\left(w_{1,j}, \ldots, w_{n,j}\right) x
\end{aligned}$$

To work out the solution, let

$\boldsymbol{A}$ denote the 3×3 matrix $\boldsymbol{X}^T \boldsymbol{W} \boldsymbol{X}$. Observe that $\boldsymbol{A}$ is symmetric by definition.

$\boldsymbol{B}$ denote the 3×1 matrix $\boldsymbol{X}^T \boldsymbol{W} \boldsymbol{y}$.

Then, we have:

$$
\begin{aligned}
\boldsymbol{A}_{1,1} &= \sum_j \sum_i w_{i,j} && = \sum_i \left(\sum_j \frac{p_{i,j}}{\sigma_j^2} \right) = \sum_i s_{i,0} \\
\boldsymbol{A}_{1,2} &= \sum_j \sum_i w_{i,j} \mu_j && = \sum_i \left(\sum_j \frac{p_{i,j}}{\sigma_j^2} \mu_j \right) = \sum_i s_{i,1} \\
\boldsymbol{A}_{1,3} &= \sum_j \sum_i w_{i,j} t_i && = \sum_i t_i \left(\sum_j \frac{p_{i,j}}{\sigma_j^2} \right) = \sum_i t_i s_{i,0} \\
\boldsymbol{A}_{2,1} &= \boldsymbol{A}_{1,2} \\
\boldsymbol{A}_{2,2} &= \sum_j \sum_i w_{i,j} \mu_j^2 && = \sum_i \left(\sum_j \frac{p_{i,j}}{\sigma_j^2} \mu_j^2 \right) = \sum_i s_{i,2} \\
\boldsymbol{A}_{2,3} &= \sum_j \sum_i w_{i,j} \mu_j t_i && = \sum_i t_i \left(\sum_j \frac{p_{i,j}}{\sigma_j^2} \mu_j \right) = \sum_i t_i s_{i,1} \\
\boldsymbol{A}_{3,1} &= \boldsymbol{A}_{1,3} \\
\boldsymbol{A}_{3,2} &= \boldsymbol{A}_{2,3} \\
\boldsymbol{A}_{3,3} &= \sum_j \sum_i w_{i,j} t_i^2 && = \sum_i t_i^2 \left(\sum_j \frac{p_{i,j}}{\sigma_j^2} \right) = \sum_i t_i^2 s_{i,0} \\
\boldsymbol{B}_{1} &= \sum_j \sum_i w_{i,j} x_i && = \sum_i x_i \left(\sum_j \frac{p_{i,j}}{\sigma_j^2} \right) = \sum_i x_i s_{i,0} \\
\boldsymbol{B}_{2} &= \sum_j \sum_i w_{i,j} \mu_j x_i && = \sum_i x_i \left(\sum_j \frac{p_{i,j}}{\sigma_j^2} \mu_j \right) = \sum_i x_i s_{i,1} \\
\boldsymbol{B}_{3} &= \sum_j \sum_i w_{i,j} t_i x_i && = \sum_i t_i x_i \left(\sum_j \frac{p_{i,j}}{\sigma_j^2} \right) = \sum_i t_i x_i s_{i,0}
\end{aligned}
$$

where $s_{i,k} := \sum_j \frac{p_{i,j}}{\sigma_j^2} \mu_j^k$. Note that, in the special case when $p_{i,j} = \delta_{j,j(i)}$ for some $j(i)$, all sums over j can be replaced by single terms.

We solve this system "by hand" using Gaussian elimination as follows. We give up in all cases when the system is found to be singular. We know that $A_{1,1} > 0$, so we first compute:

$$
[\boldsymbol{A}'|\boldsymbol{B}'] = \left(\begin{array}{ccc|c} \boldsymbol{A}_{1,1} & \boldsymbol{A}_{1,2} & \boldsymbol{A}_{1,3} & \boldsymbol{B}_1 \\ 0 & \boldsymbol{A}_{2,2} - \boldsymbol{A}_{1,2} \left(\boldsymbol{A}_{2,1} / \boldsymbol{A}_{1,1} \right) & \boldsymbol{A}_{2,3} - \boldsymbol{A}_{1,3} \left(\boldsymbol{A}_{2,1} / \boldsymbol{A}_{1,1} \right) & \boldsymbol{B}_2 - \boldsymbol{B}_1 \left(\boldsymbol{A}_{2,1} / \boldsymbol{A}_{1,1} \right) \\ 0 & \boldsymbol{A}_{3,2} - \boldsymbol{A}_{1,2} \left(\boldsymbol{A}_{3,1} / \boldsymbol{A}_{1,1} \right) & \boldsymbol{A}_{3,3} - \boldsymbol{A}_{1,3} \left(\boldsymbol{A}_{3,1} / \boldsymbol{A}_{1,1} \right) & \boldsymbol{B}_3 - \boldsymbol{B}_1 \left(\boldsymbol{A}_{3,1} / \boldsymbol{A}_{1,1} \right) \end{array} \right)
$$

Case analysis:

1. $A'_{2,2} \neq 0$. Let:

$$
[\boldsymbol{A}''|\boldsymbol{B}''] = \left(\begin{array}{ccc|c} \boldsymbol{A}'_{1,1} & \boldsymbol{A}'_{1,2} & \boldsymbol{A}'_{1,3} & \boldsymbol{B}'_1 \\ 0 & \boldsymbol{A}'_{2,2} & \boldsymbol{A}'_{2,3} & \boldsymbol{B}'_2 \\ 0 & 0 & \boldsymbol{A}'_{3,3} - \boldsymbol{A}'_{2,3} \left(\boldsymbol{A}'_{3,2} / \boldsymbol{A}'_{2,2} \right) & \boldsymbol{B}'_3 - \boldsymbol{B}'_2 \left(\boldsymbol{A}'_{3,2} / \boldsymbol{A}'_{2,2} \right) \end{array} \right)
$$

We consider subcases:

(a) $A''_{3,3} \neq 0$. We have a unique solution:

$$\begin{aligned}
\hat{c} &= \boldsymbol{B}''_3 / \boldsymbol{A}''_{3,3} \\
\hat{b} &= \left(\boldsymbol{B}'_2 - \boldsymbol{A}'_{2,3}\hat{c}\right) / \boldsymbol{A}'_{2,2} \\
\hat{a} &= \left(\boldsymbol{B}_1 - \boldsymbol{A}_{1,2}\hat{b} - \boldsymbol{A}_{1,3}\hat{c}\right) / \boldsymbol{A}_{1,1}
\end{aligned}$$

(b) $A''_{3,3} = 0$. The system is singluar, we give up.

2. $A'_{2,2} = 0$ and $A'_{3,2} \neq 0$. We consider subcases:

(a) $A'_{2,3} \neq 0$. We have a unique solution:

$$\begin{aligned}
\hat{c} &= \boldsymbol{B}'_2 / \boldsymbol{A}'_{2,3} \\
\hat{b} &= \left(\boldsymbol{B}'_3 - \boldsymbol{A}'_{3,3}\hat{c}\right) / \boldsymbol{A}'_{3,2} \\
\hat{a} &= \left(\boldsymbol{B}_1 - \boldsymbol{A}_{1,2}\hat{b} - \boldsymbol{A}_{1,3}\hat{c}\right) / \boldsymbol{A}_{1,1}
\end{aligned}$$

(b) $A'_{2,3} = 0$. The system is singluar, we give up.

3. $A'_{2,2} = 0$ and $A'_{3,2} = 0$. The system is singluar, we give up.

3.1.2 Second Step

In the previous section, we computed $(\hat{a}, \hat{b}, \hat{c})$ that maximize $-\sum_{i,j} w_{i,j} \left(x_i - a - b\mu_j - ct_i\right)^2$. Let $\alpha := \sum_{i,j} w_{i,j} \left(x_i - \hat{a} - \hat{b}\mu_j - \hat{c}t_i\right)^2$. Now, we want to compute

$$\hat{d} = \mathrm{argmax}_d - \frac{\alpha}{d} - n \log d$$

By taking the derivative with respect to d and setting it to 0, we obtain:

$$\begin{aligned}
\hat{d} = \frac{\alpha}{n} &= \frac{\sum_{i,j} w_{i,j} \left(x_i - \hat{a} - \hat{b}\mu_j - \hat{c}t_i\right)^2}{n} \\
&= \frac{1}{n} \sum_{i,j} w_{i,j} \left(x_i^2 + \hat{a}^2 + \hat{b}^2\mu_j^2 + \hat{c}^2 t_i^2 - 2\hat{a}x_i - 2\hat{b}x_i\mu_j - 2\hat{c}t_i x_i + 2\hat{a}\hat{b}\mu_j + 2\hat{a}\hat{c}t_i + 2\hat{b}\hat{c}t_i\mu_j\right) \\
&= \frac{1}{n} \Bigg(\left(\sum_i x_i^2 s_{i,0}\right) + \hat{a}^2 \sum_i s_{i,0} + \hat{b}^2 \sum_i s_{i,2} + \hat{c}^2 \sum_i t_i^2 s_{i,0} \\
&\qquad - 2\hat{a} \sum_i x_i s_{i,0} - 2\hat{b} \sum_i x_i s_{i,1} - 2\hat{c} \sum_i x_i t_i s_{i,0} \\
&\qquad + 2\hat{a}\hat{b} \sum_i s_{i,1} + 2\hat{a}\hat{c} \sum_i t_i s_{i,0} + 2\hat{b}\hat{c} \sum_i t_i s_{i,1} \Bigg) \\
&= \frac{1}{n} \Bigg(\left(\sum_i x_i^2 s_{i,0}\right) + \hat{a}^2 A_{1,1} + \hat{b}^2 A_{2,2} + \hat{c}^2 A_{3,3} \\
&\qquad - 2\hat{a}B_1 - 2\hat{b}B_2 - 2\hat{c}B_3 \\
&\qquad + 2\hat{a}\hat{b}A_{1,2} + 2\hat{a}\hat{c}A_{1,3} + 2\hat{b}\hat{c}A_{2,3} \Bigg)
\end{aligned}$$

3.2 Inverse Gaussian Parameters

To model the event stdv-s, we wany to compute the scaling parameters (v, u) that maximize the log likelihood of the observed measurements:

$$\begin{aligned}\log g_e &= \sum_{i,j} p_{i,j} \log g_{i,j} \\ &= \frac{1}{2}\sum_{i,j} p_{i,j}\left(\log \lambda'_j - \log 2\pi - 3\log y_i - \frac{\lambda'_j (y_i - \eta'_j)^2}{y_i \eta'^2_j}\right) \\ &= \frac{1}{2}\sum_{i,j} p_{i,j}\left(\log u\lambda_j - \log 2\pi - 3\log y_i - \frac{u\lambda_j (y_i - v\eta_j)^2}{y_i v^2 \eta^2_j}\right).\end{aligned}$$

Where:

$\lambda_j := \frac{\eta_j^3}{\gamma_j^2}$.

$\eta'_j := v\eta_j$.

$\lambda'_j := u\lambda_j$.

We first update v by solving $\frac{\partial \log g_e}{\partial v}(u, v) = 0$:

$$\begin{aligned}\frac{\partial \log g_e}{\partial v}(u, v) &= \frac{1}{2}\sum_{i,j} -p_{i,j}\frac{u\lambda_j}{y_i \eta_j^2} \cdot 2\left(\frac{y_i}{v} - \eta_j\right) \cdot \frac{-y_i}{v^2} \\ &= \frac{u}{v^3}\sum_{i,j} p_{i,j}\lambda_j \frac{y_i - v\eta_j}{\eta_j^2} \\ &= \frac{u}{v^3}\sum_{i,j} p_{i,j}\lambda_j \left(\frac{y_i}{\eta_j^2} - v\frac{1}{\eta_j}\right).\end{aligned}$$

Setting this to 0, we obtain:

$$\begin{aligned}\hat{v} &= \frac{\sum_{i,j} p_{i,j}\lambda_j y_i/\eta_j^2}{\sum_{i,j} p_{i,j}\lambda_j/\eta_j} \\ &= \frac{\sum_i y_i \sum_j p_{i,j}\lambda_j/\eta_j^2}{\sum_i \sum_j p_{i,j}\lambda_j/\eta_j}\end{aligned}$$

Next, we compute:

$$\frac{\partial \log g_e}{\partial u}(u, v) = \frac{1}{2}\sum_{i,j} p_{i,j}\left(\frac{1}{u} - \frac{\lambda_j (y_i - v\eta_j)^2}{y_i v^2 \eta_j^2}\right).$$

We plug in $\hat{v}$, and update u by solving $\frac{\partial \log g_e}{\partial v}(u, \hat{v}) = 0$:

$$\begin{aligned}\frac{1}{\hat{u}}\left(\sum_{i,j} p_{i,j}\right) &= \sum_{i,j} p_{i,j}\lambda_j\left(\frac{y_i}{\hat{v}^2\eta_j^2} - \frac{2}{\hat{v}\eta_j} + \frac{1}{y_i}\right) \\ &= \sum_{i,j} p_{i,j}\lambda_j\left(\frac{1}{y_i} - \frac{1}{\hat{v}\eta_j}\right) \\ &= \left(\sum_i \frac{1}{y_i}\sum_j p_{i,j}\lambda_j\right) - \frac{1}{\hat{v}}\left(\sum_i\sum_j p_{i,j}\lambda_j \frac{1}{\eta_j}\right),\end{aligned}$$

where in the second last step we used the fact that $\sum_{i,j} p_{i,j}\lambda_j y_i/\eta_j^2 = \hat{v}\sum_{i,j} p_{i,j}\lambda_j/\eta_j$. Thus,

$$\hat{u} = \frac{n}{\left(\sum_i \frac{1}{y_i}\sum_j p_{i,j}\lambda_j\right) - \frac{1}{\hat{v}}\left(\sum_i\sum_j p_{i,j}\lambda_j \frac{1}{\eta_j}\right)}.$$

4 State Transition Parameters

Given kmers k_1, k_2, and state transition parameters p_{stay}, p_{skip}, the probability of the transition from k_1 to k_2 is:

$$\begin{aligned}\text{tr}(k_1,k_2) = \quad & \delta_{k_1=k_2}\cdot p_{\text{stay}} \\ & + \delta_{\text{suffix}(k_1,5)=\text{prefix}(k_2,5)}\cdot p_{\text{step}}\cdot\frac{1}{4} \\ & + \sum_{i=2}^{5}\delta_{\text{suffix}(k_1,6-i)=\text{prefix}(k_2,6-i)}\cdot p_{\text{skip1}}^{i-1}\cdot\frac{1}{4^i} \\ & + \sum_{i>5} p_{\text{skip1}}^{i-1}\cdot\frac{1}{4^6},\end{aligned}$$

where:

δ_E is the indicator function for event E.

$p_{\text{step}} := 1 - p_{\text{stay}} - p_{\text{skip}}$.

$p_{\text{skip1}} := p_{\text{skip}}/(1+p_{\text{skip}})$ is the solution of $\sum_{i\geq 1} p_{\text{skip1}} = p_{\text{skip}}$.

To train the state transtion parameters, we first identify those states/kmers k for which there is as little ambiguity as possible for transitioning from k either back to k or to one of its 4 immediate neighbours. Formally, define:

$$\begin{aligned}\text{dist}(k_1,k_2) &:= \min\{i \mid \text{suffix}(k_1,6-i) = \text{prefix}(k_2,6-i)\} \\ \text{neigh}(k) &:= \{k' \mid \text{dist}(k,k') = 1\} \\ \text{selfoverlap}(k) &:= \max\{i \mid \text{suffix}(k,i) = \text{prefix}(k,i)\}.\end{aligned}$$

With this notation, let:

$$\mathcal{K} := \{k \mid \text{selfoverlap}(k) = 0 \text{ and } \forall k' \in \text{neigh}(k), \text{selfoverlap}(k') \leq 1\}.$$

Observe that for $\forall k \in \mathcal{K}$, and $\forall k' \in \text{neigh}(k)$,

$$\begin{aligned}\text{tr}(k,k) &= p_{\text{stay}} + \sum_{i>5} p_{\text{skip1}}^{i-1}\cdot\frac{1}{4^6} \sim p_{\text{stay}} \\ \text{tr}(k,k') &= p_{\text{step}}\cdot\frac{1}{4} + \sum_{i>5} p_{\text{skip1}}^{i-1}\cdot\frac{1}{4^6} \sim p_{\text{step}}\cdot\frac{1}{4}.\end{aligned}$$

We use the standard Forward-Backward notation:

$$\begin{aligned}\text{fw}(i,k) &:= \Pr[X_1 = x_i,\ldots,X_i = x_i, S_i = k \mid \theta] \\ \text{bw}(i,k) &:= \Pr[X_{i+1} = x_{i+1},\ldots,X_n = x_n \mid S_i = k,\theta] \\ \Pr[X|\theta] &:= \sum_k \text{fw}(n,k) \\ \text{posterior}(i,k) &:= \Pr[S_i = k|X,\theta] = \frac{\text{fw}(i,k)\cdot\text{bw}(i,k)}{\Pr[X|\theta]}\end{aligned}$$

Following the Baum-Welch algorithm, let:

$$\begin{aligned}\text{joint\_prob}(i,k,k') &:= \Pr[S_i = k, S_{i+1} = k' | X, \theta] \\ &= \frac{\text{fw}(i,k) \cdot \text{tr}(k,k') \cdot \text{em}(k', i+1) \cdot \text{bw}(i+1, k')}{\Pr[X|\theta]}\end{aligned}$$

Now we compute:

$$\begin{aligned}\text{denom}(i) &:= \Pr[S_i \in \mathcal{K} | X, \theta] = \sum_{k \in \mathcal{K}} \text{posterior}(i,k) \\ \text{numer\_stay}(i) &:= \Pr[S_i \in \mathcal{K}, S_{i+1} = S_i | X, \theta] = \sum_{k \in \mathcal{K}} \text{joint\_prob}(i,k,k) \\ \text{numer\_skip}(i) &:= \Pr[S_i \in \mathcal{K}, S_{i+1} \notin \{S_i\} \cup \text{neigh}(S_i) | X, \theta] \\ &= \sum_{k \in \mathcal{K}} \left(1 - \text{joint\_prob}(i,k,k) - \sum_{k' \in \text{neigh}(k)} \text{joint\_prob}(i,k,k') \right)\end{aligned}$$

Using these quantities, we can compute the following transition parameter updates:

$$\begin{aligned}p'_{\text{stay}} &:= \frac{\sum_{i=1}^{n-1} \text{numer\_stay}(i)}{\sum_{i=1}^{n-1} \text{denom}(i)} \\ p'_{\text{skip}} &:= \frac{\sum_{i=1}^{n-1} \text{numer\_skip}(i)}{\sum_{i=1}^{n-1} \text{denom}(i)}\end{aligned}$$

References

[1] Tim Massingham, *Calibrating and training using Expectation Maximization*, Oxford Nanopore Wiki.
